# Multivariate Pattern Analysis of Perfusion and Oxygenation Impairment in Asymptomatic Carotid Artery Stenosis

**DOI:** 10.1111/jon.70084

**Published:** 2025-09-14

**Authors:** Jan Kufer, Christine Preibisch, Jens Göttler, Lena Schmitzer, Gabriel Hoffmann, Michael Kallmayer, Claus Zimmer, Fahmeed Hyder, Stephan Kaczmarz

**Affiliations:** ^1^ Department of Diagnostic and Interventional Neuroradiology, School of Medicine and Health, TUM Klinikum Rechts Der Isar Technical University of Munich (TUM) Munich Germany; ^2^ TUM Neuroimaging Center (TUM‐NIC) Technical University of Munich (TUM) Munich Germany; ^3^ Magnetic Resonance Research Center (MRRC), Department of Radiology and Biomedical Imaging Yale University New Haven Connecticut USA; ^4^ Athinoula A. Martinos Center for Biomedical Imaging Massachusetts General Hospital Charlestown Massachusetts USA; ^5^ Department of Neurology, School of Medicine and Health, TUM Klinikum Rechts Der Isar Technical University of Munich (TUM) Munich Germany; ^6^ München Klinik Harlaching Academic Teaching Hospital of the Ludwig‐Maximilians‐University Institute for Radiology and Neuroradiology Munich Germany; ^7^ Department of Vascular and Endovascular Surgery, School of Medicine and Health, TUM Klinikum Rechts Der Isar Technical University of Munich (TUM) Munich Germany; ^8^ Philips GmbH Market DACH Hamburg Germany

**Keywords:** arterial spin labeling, asymptomatic internal carotid artery stenosis, cerebral blood flow, magnetic resonance imaging, oxygen extraction fraction, scaled subprofile model principal component analysis

## Abstract

**Background and Purpose:**

Hemodynamic impairment may contribute to stroke risk and cognitive decline in asymptomatic internal carotid artery stenosis (ICAS). Therefore, multimodal MRI‐based quantification of hemodynamic impairment could inform improved treatment decisions. While gross interhemispheric hemodynamic imbalances have been reported in ICAS, identifying more spatially resolved patterns of disease‐related alterations may be promising to harness the full potential of hemodynamic MRI.

**Methods:**

In this feasibility study, we investigated the spatial topography of ICAS‐related impairments by applying scaled subprofile model principal component analysis (SSM‐PCA) to cerebral blood flow (CBF), relative oxygen extraction fraction (rOEF), and oxygen extraction capacity (OEF^max^) data of 21 unilateral ICAS patients and 25 healthy controls (HC).

**Results:**

We found spatially extended, partly overlapping disease‐related patterns for CBF and OEF^max^, but not rOEF. CBF (area under the curve [AUC] = 0.95) but not OEF^max^ (AUC = 0.72) SSM‐PCA scores distinguished ICAS patients and HC better than interhemispheric lateralizations (AUC = 0.75/0.73). SSM‐PCA scores were only partly explained by interhemispheric lateralization (*R* ^2^ = −0.27/0.38), indicating complementary information. Critically, ICAS patients with higher OEF^max^ SSM‐PCA scores (*z* ≥ 1) demonstrated higher stenotic degrees and lower cognitive performance (*p* < 0.05) without differing in interhemispheric lateralization (*p* > 0.05).

**Conclusions:**

We demonstrated the feasibility of SSM‐PCA in ICAS and obtained novel insights into complex hemodynamic impairment patterns and their association with cognitive function.

## 1 Introduction

Internal carotid artery stenosis (ICAS) has been linked to stroke risk [1] and cognitive decline [2], even in patients termed “asymptomatic.” MR imaging of hemodynamic function has gained interest for its potential to uncover novel imaging phenotypes related to adverse clinical trajectories, aiming to improve treatment decisions. Pathomechanisms underlying cognitive deterioration in asymptomatic ICAS are still debated but could include chronic hemodynamic insufficiency [2], where reduced cerebral blood flow (CBF) may limit cerebral oxygen metabolism. Oxygen metabolism is linked to CBF and the oxygen extraction fraction (OEF), that is, the fractional utilization of oxygen supplied by cerebral arteries. While high OEF is typically thought to indicate misery perfusion and tissue at risk [3], necessary OEF increases may also be attenuated by capillary dysfunction, which is characterized by increased capillary transit time heterogeneity, previously shown to impose an upper limit on OEF, that is, oxygen extraction capacity (OEF^max^) [4]. Several studies demonstrated hemodynamic impairments in ICAS [5, 6, 7], including ipsilateral CBF reductions and mixed findings for OEF [5, 8]. Importantly, most studies focused on gross interhemispheric imbalances, that is, lateralization based on parameter averages within entire hemispheres, vascular territories [9], borderzones [10], or individualized regions of increased time to peak [7]. However, understanding the full complexity of hemodynamic alterations in ICAS may benefit from data‐driven and more spatially resolved analysis strategies that allow for consideration of regional differences in hemodynamic impairment and identification of potentially diffuse contributions. These effects may arise from variable baseline demand for oxygen across cortical regions and/or regional differences in vascular vulnerability, alone or synergistically—for example, with known age‐related patterns of widespread perfusion changes [11].

A data‐driven analysis strategy that may yield deeper insights into ICAS‐induced hemodynamic changes is scaled subprofile model principal component analysis (SSM‐PCA) [12, 13, 14]. SSM‐PCA is a feature reduction technique that has been applied to a range of pathologies and neuroimaging data, including PET [12, 15] and arterial spin labeling (ASL) [11, 14], but remained unexplored in ICAS. SSM‐PCA decomposes the subject‐by‐image space into eigenimages representing orthogonal sources of variance, potentially revealing informative disease‐related features in a data‐driven fashion.

In this study, we investigated the feasibility of SSM‐PCA to obtain novel insights into potentially complex patterns of perfusion and oxygenation impairments in ICAS patients. We hypothesized the existence of more specific disease‐related spatial covariance patterns of characteristic perfusion and oxygenation changes containing information beyond known interhemispheric lateralization. Additionally, we hypothesized differences in carotid artery morphology (i.e., stenotic degree), white matter hyperintensity (WMH) burden per the Fazekas score [16], and cognitive function according to Mini‐Mental State Examination (MMSE) scores in patients with higher versus lower (i.e., comparable to healthy controls [HC]) SSM‐PCA pattern expression scores.

## 2 Methods

### 2.1 Participants

We analyzed data from an MRI study in 29 patients with asymptomatic (no previous stroke/TIA), unilateral, extracranial, high‐grade (≥70% according to North American Symptomatic Carotid Endarterectomy Trial criteria [17]) ICAS and 30 age‐matched HC. The Klinikum rechts der Isar Medical Ethical Board approved the study. All subjects provided written informed consent before participation. We excluded one right‐sided patient with <70% stenosis, five subjects with severe MRI artifacts (e.g., motion, failed labeling in pseudocontinuous ASL [pCASL]; one left‐sided, one right‐sided, three HC), and seven subjects with a different pCASL sequence (two‐dimensional readout and/or different background suppression pulses; three left‐sided, two right‐sided, two HC), resulting in 21 patients (15 right‐sided/six left‐sided, mean age: 70.3 ± 7.4 years) and 25 HC (mean age: 70.4 ± 4.8 years).

### 2.2 Data Acquisition and Image Preprocessing

**FIGURE 1:**
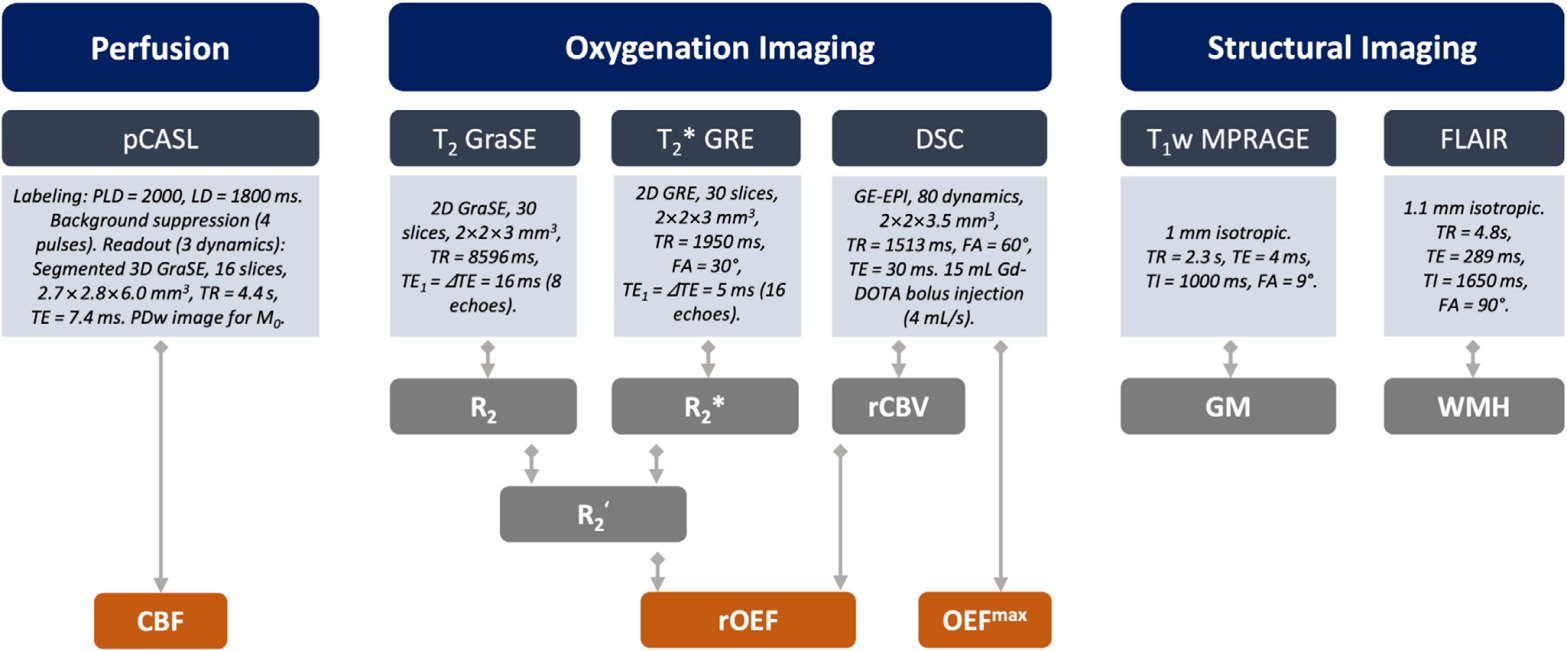
Overview of the MR protocol and sequence parameters. DSC, dynamic susceptibility contrast; FA, flip angle; FLAIR, fluid attenuated inversion recovery; GE‐EPI, gradient echo‐echo planar imaging; GM, gray matter; GraSE, gradient and spin echo; GRE, gradient echo; LD, label duration; MPRAGE, magnetization‐prepared rapid gradient echo; pCASL, pseudocontinuous arterial spin labeling; PDw, proton density‐weighted; PLD, postlabeling delay; rCBV, relative cerebral blood volume; TE, echo time; TI, inversion time; TR, repetition time; WMH, white matter hyperintensities.

Subjects were scanned on a 3T Philips Ingenia Elition X MR scanner (Philips Healthcare, Best, The Netherlands) with a 32‐channel head‐receive coil. Acquired sequences and protocol parameters are summarized in Figure 1. The total scan time for these sequences amounted to 26 min and 48 s. Additionally, subjects underwent MR angiography to rule out undetected asymptomatic stenoses in HC [9]. For pCASL, we employed a longer PLD of 2000 ms to mitigate arterial transit time (ATT) artifacts, considering subjects’ age and stenosis effects in ICAS, following International Society for Magnetic Resonance in Medicine perfusion study group guidelines [18]. Processing was conducted using MATLAB (The MathWorks, Natick, MA, USA) and SPM12 (Wellcome Trust Centre for Neuroimaging, UCL, London, UK). An experienced neuroradiologist (J.G.) assigned Fazekas scores to fluid‐attenuated inversion recovery (FLAIR) images, with supplemental ratings by J.K./L.S. to assess interrater agreement per Fleiss’ kappa. CBF was quantified from pCASL data by pairwise subtraction and averaging of motion‐corrected label‐control images, using the simplified kinetic model from Alsop et al. [18] and assuming 25% signal reduction owing to background suppression [19]. Absence of ATT artifacts was confirmed by visual inspection of unsmoothed data and a spatial coefficient of variance‐based approach with a cutoff of 0.45 [5, 20]. Maps of relative oxygen extraction fraction (rOEF) were calculated according to the multiparametric quantitative blood‐oxygen‐level‐dependent (mqBOLD) model [21], which relates venous blood oxygenation to the ratio of the transverse susceptibility‐related relaxation rate (R_2_ʹ = 1/T_2_* − 1/T_2_) and deoxygenated blood volume as approximated by relative cerebral blood volume (rCBV) from dynamic susceptibility contrast (DSC) MRI. T_2_* was obtained by monoexponential fitting of multiecho gradient‐echo data after correcting for macroscopic background gradients and motion, while for T_2_ (from multiecho gradient and spin echo data), only the even echoes were fitted [21]. To exclude areas with iron deposition and strong magnetic background field gradients, volumes of interest (VOIs) with R_2_ʹ >10 Hz were excluded from the analysis, and rOEF was capped at 0.99. Parametric deconvolution of the DSC signal intensity time curve was performed according to Mouridsen et al. [22], and a mathematical model facilitated calculation of OEF^max^ maps [4]. Parameter maps were spatially normalized to Montreal Neurological Institute standard space and resliced to match the Atlas of Intrinsic Connectivity of Homotopic Areas (AICHA) [23]. The AICHA atlas was masked to include only gray matter (GM, threshold = 0.5). Mean CBF, rOEF, and OEF^max^ values were extracted from 384 atlas‐based VOIs for each subject. Left‐/right‐sided ICAS patients’ data were pooled by grouping VOI averages of left/right hemispheres into ipsi‐/contralateral averages.

### 2.3 SSM‐PCA Analysis

Following the widely used SSM‐PCA approach [11, 12, 13, 14], spatial covariance patterns were derived for each parameter by combining disease‐identifying principal components (PCs) in logistic regression models with disease status as the dependent variable. In detail, subject‐by‐VOI data matrices were log‐transformed and double‐demeaned. Following Spetsieris et al. [13], PCA was run on the subject‐by‐subject covariance matrices first. Then, the spatial covariance patterns (i.e., PCs or eigenimages) were derived by left‐multiplying the resulting eigenvectors (scaled by the square root of the associated eigenvalues) with the transpose of subject‐by‐VOI matrices. A combined disease‐related pattern was derived for each physiological parameter separately by fitting logistic regression models [14]. Predictor PC candidates were restricted to those with a squared eigenvalue amounting to at least 1.0% of the sum of squared eigenvalues and that were disease‐identifying, that is, individually yielded significantly different pattern scores for ICAS and HC per a two‐sample *t*‐test. The combination of candidate PCs with the lowest sample size–corrected Akaike information criterion (AICc) was ultimately selected. For each parameter, left‐multiplying the log‐transformed and double‐demeaned data with the respective disease‐related pattern yielded SSM‐PCA scores, which were *z*‐transformed to a mean score of 0 in HC.

To determine the stability of pattern topographies (i.e., identify VOIs with reliably positive/negative loadings), we used a bootstrapping procedure yielding confidence intervals (CIs) for VOI loadings. Subject‐by‐VOI data matrices were resampled 1000 times, and the patterns were re‐derived following the same procedure as for the original dataset. We calculated one‐sided 90% CIs and deemed PCA loadings significant in VOIs where this CI did not include zero [15]. Pattern generalizability was estimated using leave‐one‐out cross‐validation (LOOCV) [14], that is, re‐deriving CBF/OEF^max^ SSM‐PCA patterns 46 times (leaving out each sample once). The pattern was then prospectively applied to the left‐out subject's data, and a *z*‐score was calculated as before.

For comparison, we calculated an interhemispheric lateralization index between parameter averages in ipsi‐/contralateral (mean_ipsi/contra_; left/right in HC) hemispheres’ GM:

$$\mathrm{La}t_{\mathrm{GM}}=100\%\times \frac{\mathrm{mea}n_{\mathrm{ipsi}}-\mathrm{mea}n_{\mathrm{contra}}}{0.5\times (\mathrm{mea}n_{\mathrm{ipsi}}+\mathrm{mea}n_{\mathrm{contra}})}.$$

Additionally, global GM averages (mean_GM_) were calculated. All measures were standardized (*z*‐transformed) to the parameters’ HC average, analogous to the SSM‐PCA scores.

### 2.4 Statistical Analysis

Significance was assumed at *p* < 0.05, adjusted for multiple comparisons using the Benjamini–Hochberg procedure to limit the false discovery rate (denoted as *p*_FDR_ in the following). Normality was determined using Shapiro–Wilk tests. Effect sizes were obtained as Cohen's *d* (for normally distributed samples) or Cliff's *δ* (for samples not normally distributed). Sample means were compared using Welch's two‐sample *t*‐test or nonparametric Mann–Whitney *U* tests. Paired comparisons were conducted using paired *t*‐tests or Wilcoxon signed‐rank tests. Proportions of binary measures were compared using Fisher's exact tests. Area‐under‐the‐curve (AUC) comparisons employed DeLong's test as implemented by Sun and Xu [24]. First, we compared demographic and clinical characteristics between ICAS and HC (Table 1). Second, we analyzed how SSM‐PCA patterns distinguished patients from HC in comparison with other hemodynamic measures (Lat_GM_, mean_ipsi_, and mean_GM_) by testing for between‐group differences of *z*‐scores for CBF and OEF^max^ SSM‐PCA expression, Lat_GM_, mean_ipsi_, and mean_GM_. We also conducted paired tests to assess whether *z*‐scores between disease‐related measures differed significantly within patients. Furthermore, for parameters distinguishing ICAS from HC, we obtained AUC values to quantify their combined sensitivity/specificity. LOOCV scores were also included in this comparison to establish whether prospective performance was comparable to that of the SSM‐PCA scores derived from the full sample. Third, we analyzed interrelations between SSM‐PCA scores, Lat_GM_, mean_GM_, and mean_ipsi_ of CBF and OEF^max^. We computed *R* ^2^ values for pairwise Pearson correlations, both within and across CBF/OEF^max^, and separately for ICAS/HC. Lastly, we investigated whether patients with higher pattern scores differed from those with lower SSM‐PCA scores in terms of stenotic degree, Fazekas score, MMSE, or Lat_GM_. We split patients into two subgroups with greater (*z* ≥ 1) and more modest (*z* < 1) hemodynamic impairment in terms of SSM‐PCA pattern expression for CBF or OEF^max^. The threshold of *z* = 1 corresponded to the median SSM‐PCA score of patients across CBF/OEF^max^ patterns.

**TABLE 1:** Participant demographics.

	Controls (*n* = 25)	Patients (n = 21)	*p*‐value
Age, years	70.4 ± 4.8	70.3 ± 7.4	0.97
Sex female, *n* (%)	14 (56)	8 (38)	0.25
BMI, kg/m^2^	26.2 ± 3.9	26.0 ± 4.5	0.90
Stenotic degree, % NASCET	Not applicable	80 (IQR: 12.5)	Not applicable
Left‐/right‐sided stenosis, *n*	Not applicable	6/15	Not applicable
Fazekas score of WMH	1 (IQR: 2)	2 (IQR: 1)	0.02^a^
Hypertension, *n* (%)	14 (56%)	18 (86%)	0.05
BP systolic/diastolic, mmHg	141 ± 20/84 ± 7	159 ± 22/87 ± 11	0.01^a^/0.22
CHD, *n* (%)	1 (4%)	5 (24%)	0.08
PAD, *n* (%)	4 (16%)	4 (19%)	1.00
Diabetes, *n* (%)	2 (8%)	4 (19%)	0.39
Smoker, *n* (%)	8 (32%)	11 (52%)	0.23
Pack years in smokers	16.4 ± 14.3	38.5 ± 18.4	0.009^a^
MMSE	29 (IQR: 2)	29 (IQR: 3)	0.73
TMT‐A, s	40.0 (IQR: 22.3)	41.6 (IQR: 20.4)	0.55
TMT‐B, s	101.2 (IQR: 40.0)	108.1 (IQR: 101.5)	0.11
LBT, abs. % deviation	3.1 ± 1.9	2.3 ± 2.2	0.11
BDI	8.1 ± 4.6	8.7 ± 10.1	0.41
STAI	33.5 ± 9.3	38.3 ± 11.0	0.14

*Note*: All data are presented as mean ± standard deviation or median (interquartile range [IQR]). Count data are presented as number (*n*) and % within subgroup.

Abbreviations: BDI, Beck's Depression Inventory; BMI, body mass index; BP, blood pressure; CHD, coronary heart disease; LBT, Line Bisection Test; MMSE, Mini‐Mental State Examination; NASCET, North American Symptomatic Carotid Endarterectomy Trial; PAD, peripheral artery occlusive disease; STAI, State‐Trait Anxiety Inventory; TMT‐A/‐B, Trail Making Test A/B; WMH, white matter hyperintensities.

^a^ Not significant after correcting for multiple comparisons.

## 3 Results

### 3.1 Participant Demographics

Table 1 summarizes demographic and clinical characteristics of ICAS patients and HC. Systolic blood pressure, pack years in smokers, and Fazekas scores tended to be higher in ICAS (unadjusted *p* < 0.05), but these differences did not reach significance after adjusting for multiple comparisons. Substantial agreement between raters’ Fazekas scores was observed (Fleiss’ kappa = 0.65). Both groups performed similarly on all neuropsychiatric tests (*p* > 0.05).

### 3.2 Spatial Topographies of Hemodynamic Changes Revealed by SSM‐PCA

**FIGURE 2:**
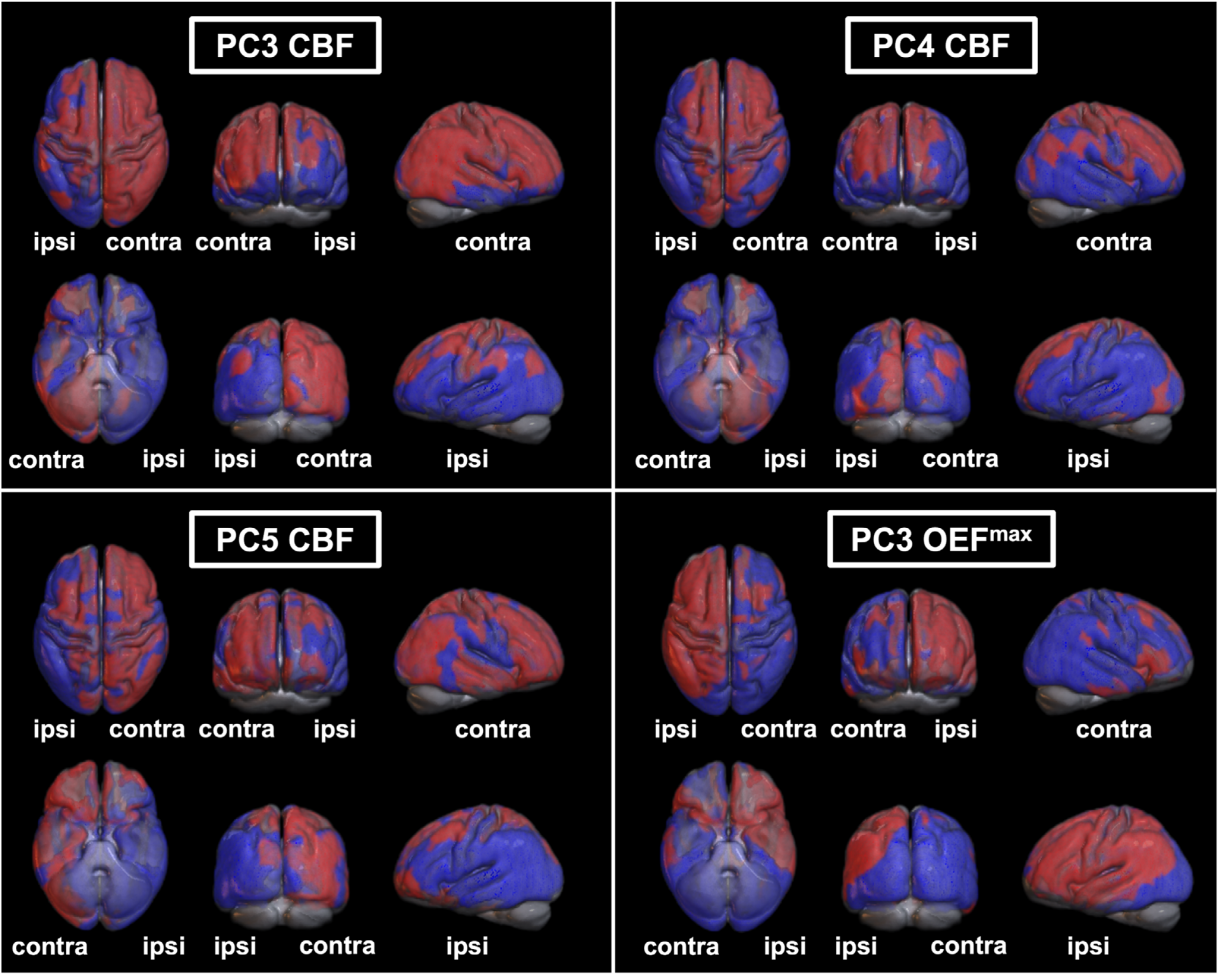
Topography of individual principal components (PCs) distinguishing ICAS from HC. Three‐dimensional renderings of binarized PCs overlaid on the mean T_1_‐weighted MRI of all participants. Ipsi‐/contralateral hemispheres are marked accordingly. Regions with positive loadings are displayed in red, while regions with negative loadings are shown in blue. For CBF, PC3 (top left), PC4 (top right), and PC5 (bottom left) yielded significantly different scores between ICAS and HC, whereas only PC3 separated ICAS from HC for OEF^max^.

**FIGURE 3:**
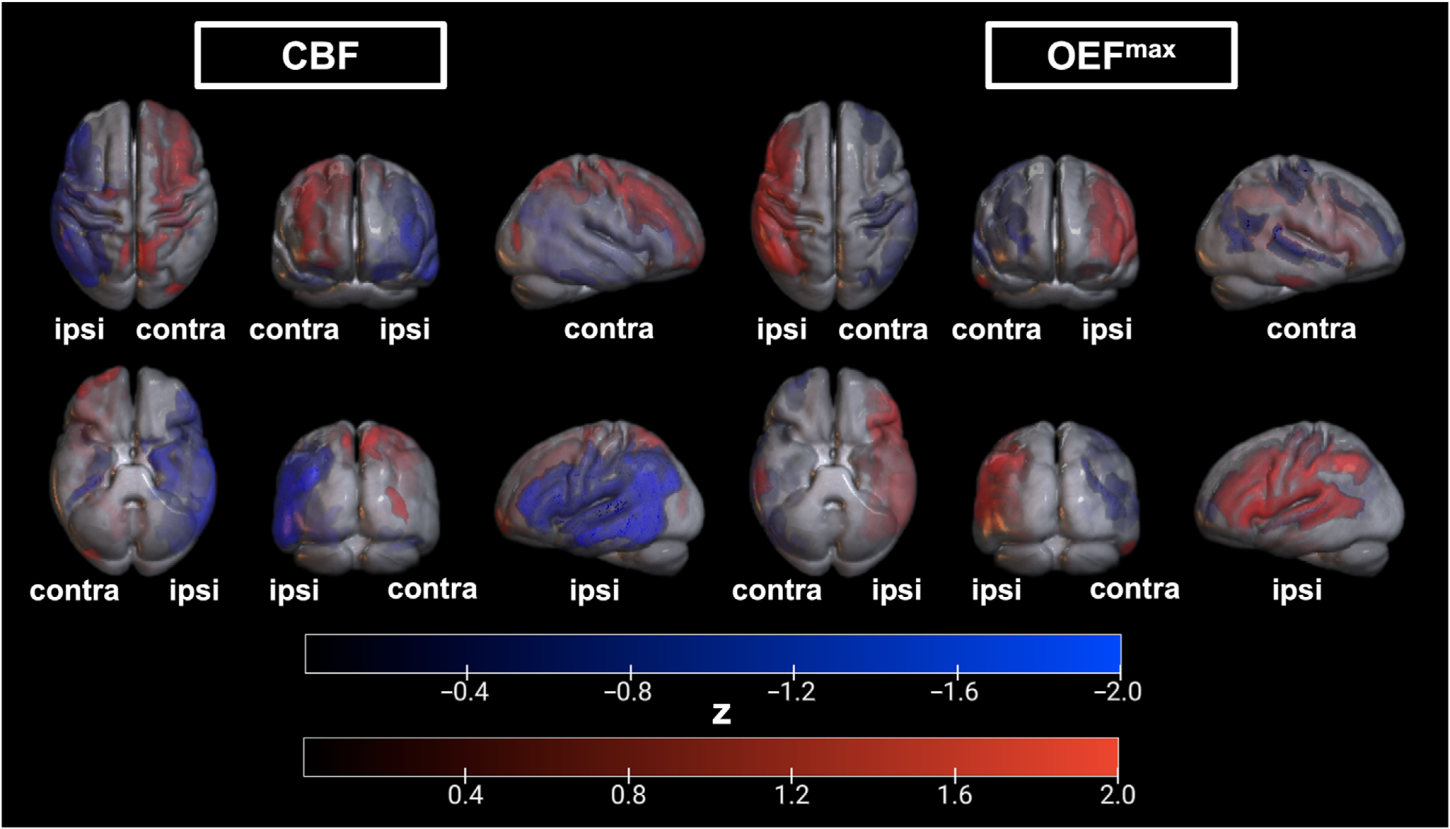
Combined spatial patterns of characteristic CBF and OEF^max^ changes in ICAS. Images depict stable areas of positive (red) and negative loadings (blue) from the combined ICAS‐related CBF (left) and OEF^max^ (right) patterns that survived bootstrapping. Ipsi‐/contralateral hemispheres are marked accordingly. Note topographical similarities between CBF/OEF^max^ patterns (but with inverted loading signs) visually involving the ipsilateral middle cerebral artery territory and contralateral borderzones.

Spatial topographies of individual PCs are displayed in Figure 2. PC3, PC4, and PC5 for CBF, but only PC3 for OEF^max^, differentiated patients from HC (*p* < 0.05). From these PCs, disease‐related spatial patterns for CBF and OEF^max^ were determined as the combinations with the lowest AICc. For CBF, this pattern consisted of the combination of PC3, PC4, and PC5, explaining 9.2%, 8.4%, and 6.3% of the variance, respectively, or 23.9% of all variance in the data. For OEF^max^, only PC3, explaining 10.8% of variance, fulfilled the selection criteria (share in squared eigenvalues ≥1% and significantly different SSM‐PCA scores between ICAS/HC). For rOEF, no PC was related to ICAS per the above‐specified criteria. Spatial topographies of combined hemodynamic patterns for CBF and OEF^max^ are shown in Figure 3, displaying only areas of stable positive/negative loadings. For both, multiple areas survived bootstrapping, and SSM‐PCA patterns showed extensive regional overlap between CBF/OEF^max^. Most prominent changes included negative (CBF)/positive (OEF^max^) loadings, mostly within the ipsilateral middle cerebral artery (MCA) territory. Contralaterally, areas of surviving positive (CBF) and negative loadings (OEF^max^) were observed at locations where the borderzone regions between the anterior cerebral artery (ACA)/MCA territories and the posterior cerebral artery/MCA territories would be expected. The CBF pattern also included bilateral, positive loadings comprising the precentral sulcus and precuneus regions.

### 3.3 Sensitivity/Specificity of Hemodynamic Measures to ICAS‐Related Impairments

**FIGURE 4:**
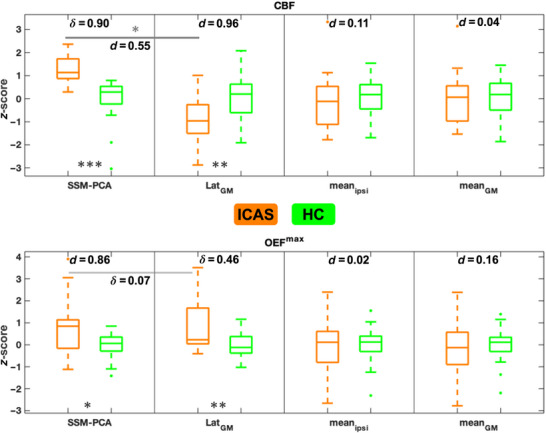
Comparison of standardized CBF and OEF^max^ measures between ICAS and HC. Standardized (*z*‐transformed, centered on HC averages) SSM‐PCA pattern scores, interhemispheric parameter lateralization (Lat_GM_), and ipsilateral (mean_ipsi_) and global (mean_GM_) averages in gray matter are compared between ICAS (orange boxplots) and HC (green boxplots). Asterisks indicate significant differences (*p < 0.05,**p < 0.01,***p < 0.001; uncorrected *p*‐values) that all survived adjustment for multiple comparisons. Effect sizes are shown as Cliff's delta (*δ*) or Cohen's *d*. Solid gray lines indicate within‐ICAS comparisons between disease‐related parameters (i.e., SSM‐PCA and Lat_GM_).

Results of group‐level comparisons between ICAS and HC for *z*‐scores of SSM‐PCA expression, Lat_GM_, mean_ipsi_, and mean_GM_ in CBF and OEF^max^ are shown in Figure 4. SSM‐PCA pattern scores were significantly higher in patients than in HC, more so for CBF (Cliff's *δ* = 0.90; *p*_FDR_ < 0.001) than OEF^max^ (Cohen's *d* = 0.86; *p*_FDR_ = 0.03). Lat_GM_ of CBF and OEF^max^ was the only other measure with elevated absolute *z*‐scores in ICAS. CBF was relatively reduced toward the affected side (Cohen's *d* = 0.96; *p*_FDR_ = 0.006, negative *z*‐scores), whereas the opposite was the case for OEF^max^ (Cliff's *δ* = 0.46; *p*_FDR_ = 0.03). In patients, CBF but not OEF^max^ SSM‐PCA scores were more strongly increased than Lat_GM_ absolute *z*‐scores (Cohen's *d* = 0.55; *p*_FDR _< 0.05). Neither mean_GM_ nor mean_ipsi_ of either CBF or OEF^max^ differed between ICAS and HC (Cohen's *d* < 0.20; *p* > 0.05). The CBF SSM‐PCA pattern also identified ICAS‐related impairments with greater combined sensitivity/specificity (AUC = 0.95) than the OEF^max^ pattern (AUC = 0.72). Predictive performance of the SSM‐PCA OEF^max^ pattern upon prospective application was comparable to that in the original sample (AUC = 0.67 for LOOCV, *p* = 0.13 in DeLong's test), while for CBF, classification performance from LOOCV was slightly lower than for the original sample, but still very high (AUC = 0.84 for LOOCV, *p* = 0.03). In addition, for CBF but not OEF^max^, the SSM‐PCA pattern provided significantly better discrimination between ICAS and HC than Lat_GM_ (AUC = 0.75, *p* = 0.01).

### 3.4 Associations Between SSM‐PCA Scores, Parameter Lateralization, and Absolute Averages

**FIGURE 5:**
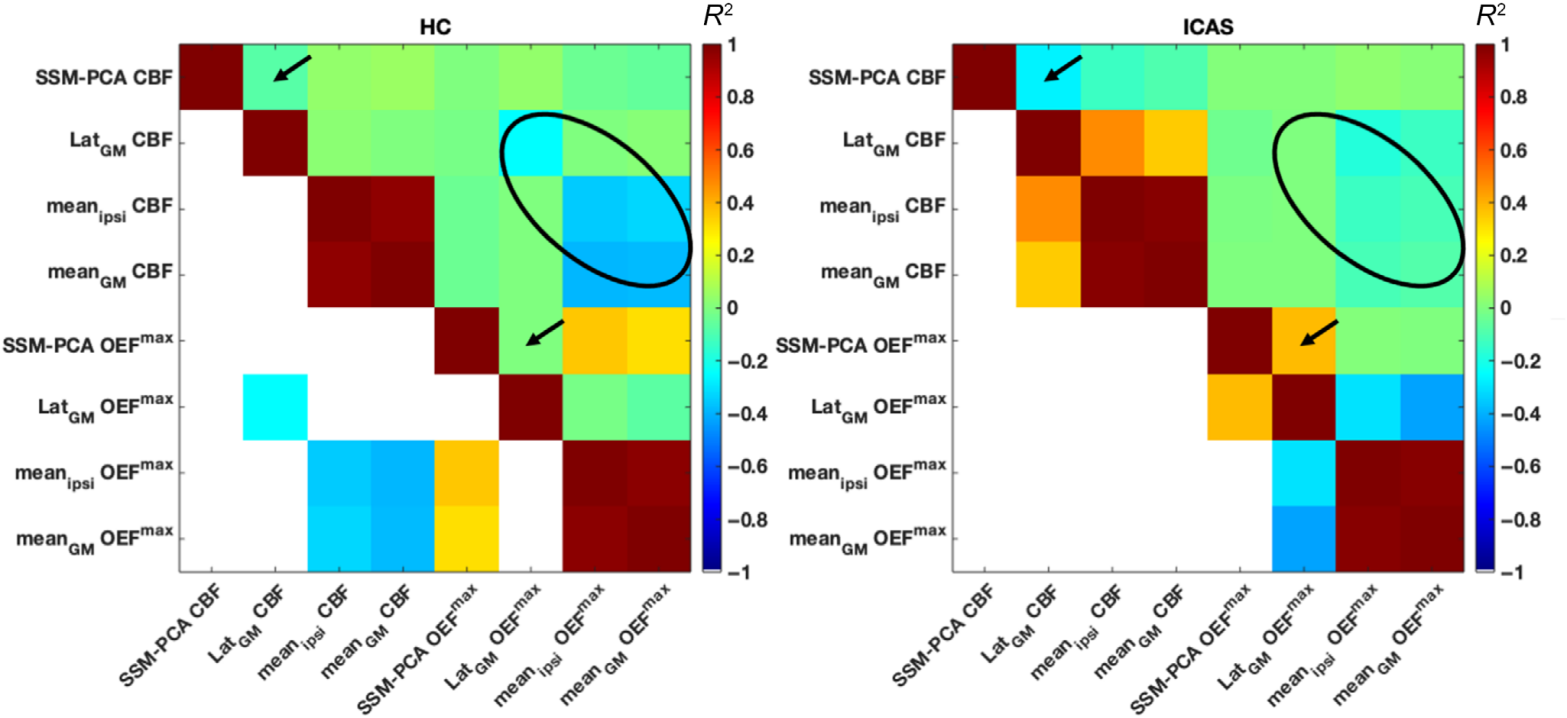
Correlation matrices within HC (left) and ICAS (right) for parameters compared in Figure 4. Signed *R* ^2^ values with red colors indicate positive and blue colors negative associations. All *R* ^2^ values are shown above the respective autoregression lines, whereas only *R* ^2^ values of associations remaining statistically significant when adjusting for multiple comparisons are shown below the autoregression line. Note modest relationships between SSM‐PCA and interhemispheric lateralization in gray matter (Lat_GM_, black arrows) in patients, indicating complementary information. In HC, there was no association between SSM‐PCA and Lat_GM_. For OEF^max^, SSM‐PCA scores in HC, but not patients, were associated with ipsilateral (mean_ipsi_) and whole brain (mean_GM_) mean gray matter values. Moderate inverse associations between corresponding CBF/OEF^max^ measures (i.e., Lat_GM_, mean_ipsi_, and mean_GM_) across participants can be appreciated in HC but appear diminished in ICAS (black circles).

Pearson correlation *R* ^2^ values for all hemodynamic measures are displayed in Figure 5, separately for ICAS and HC. CBF and OEF^max^ SSM‐PCA pattern scores were uncorrelated in both patients and HC (*R* ^2^ = 0.006/−0.003, *p* = 0.75/0.78). For CBF, SSM‐PCA and Lat_GM_ were not statistically significantly correlated in HC (*p* = 0.19) but exhibited a trend for a modest correlation in ICAS (*R* ^2^ = −0.27, *p* = 0.02) that did not survive multiple comparisons correction. For OEF^max^, the association between SSM‐PCA scores and Lat_GM_ was equally stronger in patients (*R* ^2^ = 0.38, *p*_FDR_ = 0.02) than in HC (*p* = 0.79). In HC, we observed modest negative associations between CBF and OEF^max^ for Lat_GM_, mean_ipsi_, and mean_GM_, with *R* ^2^ ranging from –0.39 to –0.25 (*p*_FDR_ < 0.05), which were greatly diminished in ICAS (all *p* > 0.05).

### 3.5 Stenotic Degrees, WMH Burden, and Cognitive Function in Subgroups According to SSM‐PCA Expression

**FIGURE 6:**
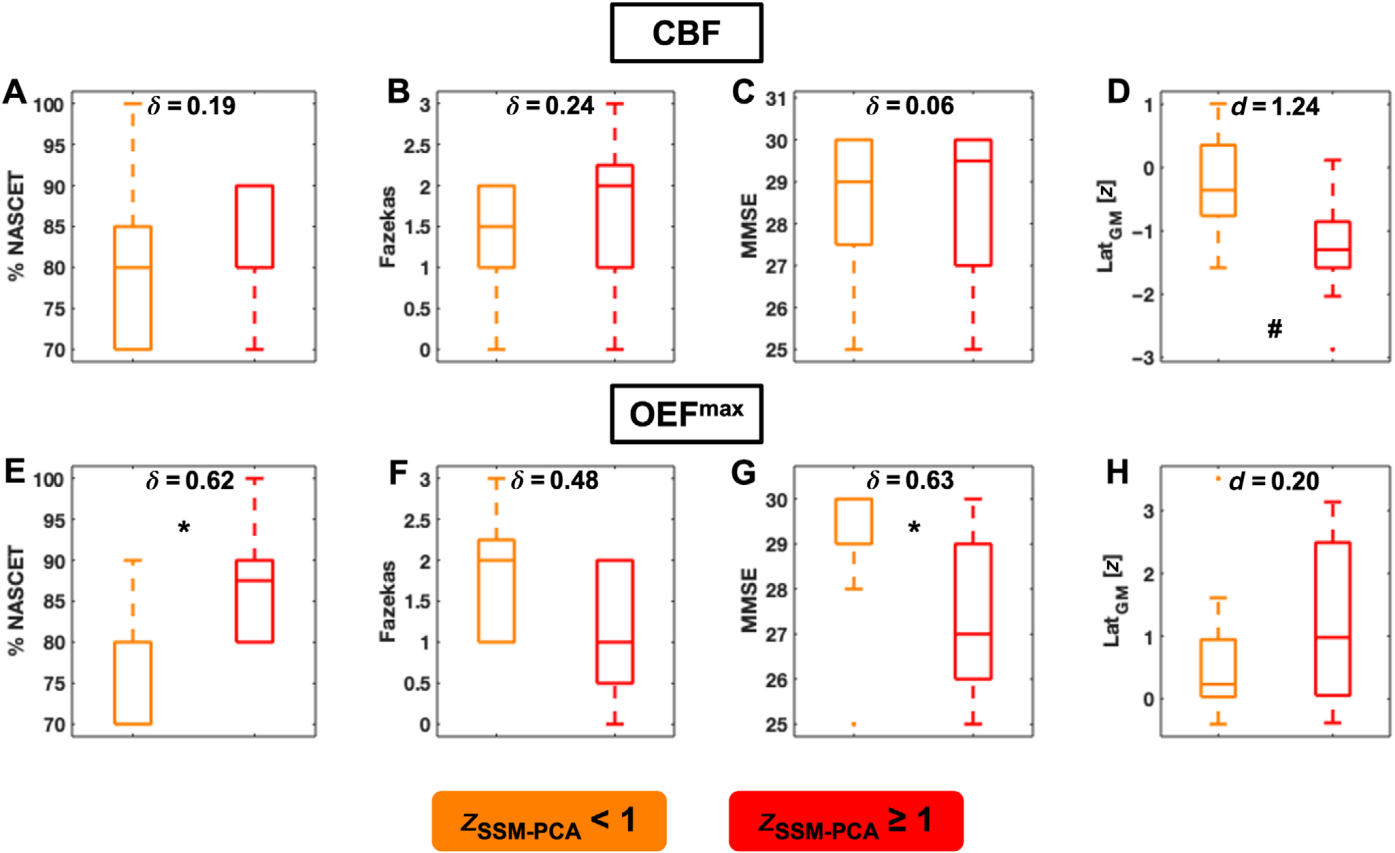
Comparison of clinical measures in ICAS subgroups of stronger and weaker SSM‐PCA pattern expression. Stenotic degree according to North American Symptomatic Carotid Endarterectomy Trial (NASCET) criteria (A, E), Fazekas scores (B, F), and cognition (Mini‐Mental State Examination [MMSE] scores; C, G) are shown for patients with higher (*z* ≥ 1, red boxplots) and lower (*z* < 1, orange boxplots) SSM‐PCA scores. For comparison, differences in interhemispheric parameter lateralization in gray matter (Lat_GM_) by SSM‐PCA subgroups were also analyzed (D, H). Statistically significant differences before adjusting for multiple comparisons (i.e., unadjusted *p* < 0.05) are marked by the # symbol, whereas those surviving correction for multiple comparisons are marked by an asterisk. Effect sizes are shown as Cliff's delta (*δ*) or Cohen's *d*.

Lastly, we compared stenotic degree, Fazekas scores, and cognition (MMSE) between patients with more severe hemodynamic impairment in terms of CBF/OEF^max^ SSM‐PCA pattern scores (*z* ≥ 1) and those with scores more similar to HC (*z* < 1; Figure 6). We observed that these patient subgroups did not differ in either of these clinical parameters when identified by their SSM‐PCA scores for the pattern of CBF changes (Cliff's *δ* < 0.25; *p* > 0.05). In contrast, high OEF^max^ pattern scores indicated ICAS patients with higher stenotic degrees (Cliff's *δ* = 0.62; *p*_FDR_ < 0.05) and subtly lower MMSE scores (Cliff's *δ* = 0.63; *p*_FDR_ < 0.05). Interestingly, this clinically more impaired subgroup did not differ in terms of either CBF or OEF^max^ lateralization (Cohen's *d* = 1.24/0.20; *p*_FDR_ > 0.05). Finally, high/low‐SSM‐PCA‐score subgroups’ WMH burden did not differ (*p*_FDR_ > 0.05) for CBF/OEF^max^ alike (Cliff's *δ* = 0.24/0.48).

## 4 Discussion

We applied SSM‐PCA to investigate spatial topographies of disease‐related changes in CBF, rOEF, and OEF^max^ and to derive novel data‐driven measures of hemodynamic impairment in asymptomatic unilateral ICAS. We found complex and characteristic spatial patterns of CBF and OEF^max^, but not rOEF, changes. These spatial covariance patterns were characterized by positive (OEF^max^) and negative (CBF) PCA loadings within the ipsilateral MCA territory, but comprised additional regions along contralateral borderzones. SSM‐PCA pattern expression in patients was only moderately correlated with Lat_GM_, suggesting overlapping and distinct information. For CBF, SSM‐PCA exhibited greater combined sensitivity/specificity than Lat_GM_, while for OEF^max^, pattern expression was more variable among patients, with *z*‐scores comparable to those of interhemispheric lateralization. However, patients exhibiting more pronounced OEF^max^ SSM‐PCA pattern expression (*z* ≥ 1) presented with elevated stenotic degrees and subtly lower cognitive performance but did not differ in terms of OEF^max^ lateralization. Overall, our findings suggest the feasibility and potential of SSM‐PCA applications in ICAS, highlighting its potential utility in terms of identifying novel phenotypes of hemodynamic impairment that, if validated in larger cohorts, may have the potential to improve our understanding of ICAS‐induced cortical hemodynamic impairment and its link to cognitive decline.

### 4.1 Spatial Topography of CBF and OEF^max^ Changes

We found complex patterns of regional CBF and OEF^max^ changes distinguishing ICAS patients from HC. Importantly, although parameters were acquired separately and patterns were derived independently, disease‐related effects were located in similar regions (Figure 3). In overlapping regions, positive CBF loadings were accompanied by negative OEF^max^ loadings and vice versa. While this could indicate compensation for hypoperfusion [3], it is unclear if this is the case, given that we did not find a similar pattern for rOEF. A lack of consistent OEF elevations in asymptomatic patients agrees with previous studies [6, 8]. Besides potential methodological limitations, OEF^max^, as the modeled maximum OEF, is distinct from OEF [4], and future work should clarify the underpinnings of OEF^max^–rOEF discrepancies. Next, most bootstrapping‐surviving areas were located within the ipsilateral MCA territory. Relatively reduced ipsilateral MCA perfusion agrees with existing studies [25]. Interestingly, however, both CBF and OEF^max^ changes spared the ipsilateral vascular borderzones between the MCA and ACA/posterior cerebral artery territories. Although previous studies reported the strongest hemodynamic impairments within borderzones [5, 25], watershed impairments may be diffuse and located in different subregions across patients, as increased within‐borderzone parameter standard deviations were reported [5]. Moreover, borderzones in asymptomatic ICAS exhibit increased spatial variability [10]; it is therefore plausible that these regions were not included in SSM‐PCA patterns, which consist of regions that are most consistently implicated across patients rather than areas with the largest effects in individual subjects. Besides MCA borderzones, the ipsilateral ACA territory was largely spared in both CBF and OEF^max^ patterns. Although the ACA is supplied by the ICA, previous studies found frequent territory shifts with ipsilateral ACA supply by retrograde collateral flow from the contralateral ACA [26]. Consequently, SSM‐PCA loadings for the ipsilateral ACA territory were small and did not survive bootstrapping. Interestingly, SSM‐PCA also revealed reproducible changes in contralateral regions for CBF and OEF^max^: These areas were clustered along the anterior and posterior MCA borderzones. We speculate that this phenomenon may be related to collateral flow. Interestingly, reported flow–metabolism uncoupling in unilateral ICAS was similarly driven by contralateral changes [9]. In addition, bilateral CBF but not OEF^max^ changes involved the precuneus, which is one of the main nodes of the default mode network (DMN) [27]. DMN changes in ICAS have been described before [28]; however, the impact of ICAS on functional connectivity remains controversial [29].

### 4.2 Sensitivity and Specificity of Different Hemodynamic Measures for ICAS‐Related Impairments

To assess the SSM‐PCA scores’ potential to serve as novel measures of ICAS‐related impairment, we compared their ability to distinguish patients from HC to Lat_GM_ and mean_ipsi_/mean_GM_ *z*‐scores. For CBF and OEF^max^, only SSM‐PCA pattern expression and Lat_GM_ *z*‐scores differed in ICAS versus HC (Figure 4). Relative hemispheric CBF reductions [7] and OEF^max^ increases [5] in asymptomatic ICAS have been reported before. In contrast, intragroup variability for absolute parameter averages appeared to be larger than the differences between ICAS/HC. This may be related to small effects, measurement noise, and known physiological variability [30]. Previous studies found ipsilateral absolute CBF reductions only in symptomatic patients or in cases of asymptomatic ICA occlusion, rather than in asymptomatic ICAS [31]. For CBF, the SSM‐PCA pattern was overall significantly more characteristic of ICAS than Lat_GM_ per AUCs, that is, observed widespread patterns of changes (including contralateral borderzones and bilateral precuneus regions) are very consistently expressed by patients and absent in HC. Thus, the CBF SSM‐PCA pattern may provide a more complete picture of ICAS‐related perfusion impairment. LOOCV AUC was significantly lower than full‐sample AUC; while this is in line with other SSM‐PCA studies [14], larger derivation samples will further mitigate overfitting (see Section 4.5). Nevertheless, the AUC of 0.84 obtained by LOOCV suggests reasonable generalizability of the CBF SSM‐PCA pattern. In contrast, SSM‐PCA and LOOCV scores for OEF^max^ did not differ significantly from those of Lat_GM_. In addition, the AUCs for all of these OEF^max^ measures were more modest than those for the CBF SSM‐PCA pattern but comparable to Lat_GM_ of CBF. This is in line with the proposition that oxygen extraction changes may be a marker of more severe ICAS‐induced hemodynamic impairment [6] and are only expected in a smaller subset of strongly affected patients.

### 4.3 Associations Between SSM‐PCA Scores, Parameter Lateralization, and Ipsilateral/Whole Brain Averages

Given that the topographies of SSM‐PCA patterns revealed both expected (i.e., relative ipsilateral CBF reduction and OEF^max^ elevation primarily within MCA‐supplied regions) and additional hemodynamic changes (contralateral borderzones, relative hyperperfusion bilaterally in precuneus regions), we were interested in comparing the SSM‐PCA scores to VOI‐based quantification of hemodynamic impairment (Lat_GM_, mean_ipsi_, mean_GM_), which we did separately for ICAS patients and HC. Most notably, SSM‐PCA scores and Lat_GM_ for CBF and OEF^max^ were uncorrelated within HC and relatively moderately correlated within ICAS patients (*R* ^2^ = −0.27/0.38, respectively), considering that SSM‐PCA scores and Lat_GM_ were derived from the same data (i.e., affected by the same noise). This highlights that SSM‐PCA patterns may be complementary hemodynamic markers with the potential to facilitate novel insights into associations of ICAS‐related hemodynamics and clinical impairment. Of note, SSM‐PCA scores of CBF and OEF^max^ were not substantially correlated with each other or any of the other hemodynamic measures (except Lat_GM_ of the same parameter within patients). Therefore, although the topographies of CBF and OEF^max^ showed notable spatial overlaps, this does not seem to imply a tight coupling of the expression of these two patterns across patients. Indeed, uncoupling of flow and oxygen metabolism in ICAS has been described before, based on mqBOLD data [9]. Along those lines, we also noted that an inverse correlation between OEF^max^ and CBF (for Lat_GM_, mean_GM_, and mean_ipsi_) in healthy subjects (i.e., flow–metabolism coupling) vanished in ICAS patients (Figure 5).

### 4.4 Stenotic Degrees, WMH Burden, and Cognitive Function in Subgroups According to SSM‐PCA Expression

Comparing patients with strong and weak SSM‐PCA pattern expression, we found that those with high OEF^max^ pattern scores presented with higher stenotic degrees and subtly reduced cognitive performance as measured by MMSE scores. While this could suggest that OEF^max^ impairment is indeed a marker of more advanced hemodynamic insufficiency (see discussion above), it is noteworthy that this association did not hold for gross interhemispheric imbalances of OEF^max^ (i.e., Lat_GM_). This underlines the potential of SSM‐PCA to uncover more complex patterns of hemodynamic status that may be needed to fully understand the possible link between cerebral vascular physiology and cognition in ICAS. Interestingly, no differences were observed between Fazekas scores in ICAS subgroups of high/low SSM‐PCA pattern expression, in line with a recent study linking reduced white matter integrity in ICAS to concomitant small vessel disease rather than hemodynamic compromise [32]. Moreover, patients with higher CBF pattern scores did not differ in either stenotic degree, WMH load, or MMSE. For CBF, SSM‐PCA appears to have selected a pattern that is highly characteristic of ICAS with lower within‐group variability than for OEF^max^ (Figure 4). To further investigate possible connections between patterns of hemodynamic changes and clinical impairment, future studies with larger cohort sizes, across multiple sites, and using harmonized analytical tools should directly evaluate PCs for potential associations with, for example, longitudinal cognitive decline. Then, SSM‐PCA might have the potential to inform clinical diagnosis on a single subject level, given that derived patterns can be readily applied to prospectively acquired data.

### 4.5 Limitations

First, our sample size was limited; thus, using a data‐driven approach like SSM‐PCA might entail some risk of overfitting. Following others applying SSM‐PCA in comparably sized groups [14], we applied bootstrapping and LOOCV for validation. Sample sizes were further reduced for the subgroup analyses in ICAS, shown in Figure 6, where some findings with comparably large effect sizes failed to reach statistical significance. Studies in larger cohorts will be needed to better understand these effects. Second, the pCASL and mqBOLD techniques have known methodological limitations [5]. For this particular analysis, distinguishing hypoperfusion from atrophy‐related partial volume effects might be challenging given small VOI sizes. However, a published analysis of structural data in the same data set revealed only very few significant areas of ICAS‐related local cortical atrophy [32]. Furthermore, we employed multiple steps to reduce and identify arterial transit delay effects; nevertheless, multidelay ASL sequences should be employed in future studies [33]. The mqBOLD technique is affected by biased T_2_* estimates arising from non‐heme iron and/or macroscopic background gradients, as well as T_2_ overestimation due to stimulated echoes [21]. Despite careful visual inspection and motion correction, all parameter maps might have been affected by residual influences of motion, and their impact on SSM‐PCA analyses requires further study. Third, variability of cognitive function in our sample was limited, with no cases of overt impairment (MMSE < 24), and adjustments for age, sex, or education were not feasible due to the limited cohort size.

## Conflicts of Interest

Stephan Kaczmarz was employed by Philips GmbH Market DACH, Hamburg, Germany. The remaining authors declare no conflicts of interest.

## References

[1] G. W. Petty , R. D. Brown Jr. , J. P. Whisnant , et al., “Ischemic Stroke Subtypes: A Population‐Based Study of Incidence and Risk Factors,” Stroke; A Journal of Cerebral Circulation 30 (1999): 2513–2516. 10.1161/01.str.30.12.2513 10582970

[2] H. Baradaran , A. H. Sarrami , and A. Gupta , “Asymptomatic Carotid Disease and Cognitive Impairment: What Is the Evidence?,” Frontiers in Neurology 12 (2021): 741500. 34867724 10.3389/fneur.2021.741500 PMC8636319

[3] C. P. Derdeyn , T. O. Videen , K. D. Yundt , et al., “Variability of Cerebral Blood Volume and Oxygen Extraction: Stages of Cerebral Haemodynamic Impairment Revisited,” Brain 125 (2002): 595–607. 11872616 10.1093/brain/awf047

[4] S. N. Jespersen and L. Ostergaard , “The Roles of Cerebral Blood Flow, Capillary Transit Time Heterogeneity, and Oxygen Tension in Brain Oxygenation and Metabolism,” Journal of Cerebral Blood Flow and Metabolism 32 (2012): 264–277. 22044867 10.1038/jcbfm.2011.153 PMC3272609

[5] S. Kaczmarz , J. Gottler , J. Petr , et al., “Hemodynamic Impairments Within Individual Watershed Areas in Asymptomatic Carotid Artery Stenosis by Multimodal MRI,” Journal of Cerebral Blood Flow and Metabolism 41 (2021): 380–396. 32237952 10.1177/0271678X20912364 PMC7812517

[6] W. J. Powers , C. P. Derdeyn , S. M. Fritsch , et al., “Benign Prognosis of Never‐Symptomatic Carotid Occlusion,” Neurology 54 (2000): 878–882. 10690980 10.1212/wnl.54.4.878

[7] B. Crespo Pimentel , J. Sedlacik , and J. Schröder , “Comprehensive Evaluation of Cerebral Hemodynamics and Oxygen Metabolism in Revascularization of Asymptomatic High‐Grade Carotid Stenosis,” Clinical Neuroradiology 32 (2021): 163–173. 34487195 10.1007/s00062-021-01077-3 PMC8894147

[8] J. Bouvier , O. Detante , F. Tahon , et al., “Reduced CMRO_2_ and Cerebrovascular Reserve in Patients With Severe Intracranial Arterial Stenosis: A Combined Multiparametric qBOLD Oxygenation and BOLD fMRI Study,” Human Brain Mapping 36 (2015): 695–706. 25307948 10.1002/hbm.22657 PMC6869377

[9] J. Gottler , S. Kaczmarz , M. Kallmayer , et al., “Flow‐Metabolism Uncoupling in Patients With Asymptomatic Unilateral Carotid Artery Stenosis Assessed by Multi‐Modal Magnetic Resonance Imaging,” Journal of Cerebral Blood Flow and Metabolism 39 (2019): 2132–2143. 29968499 10.1177/0271678X18783369 PMC6827123

[10] S. Kaczmarz , V. Griese , C. Preibisch , et al., “Increased Variability of Watershed Areas in Patients With High‐Grade Carotid Stenosis,” Neuroradiology 60 (2018): 311–323. 29299616 10.1007/s00234-017-1970-4

[11] N. Zhang , M. L. Gordon , Y. Ma , et al., “The Age‐Related Perfusion Pattern Measured With Arterial Spin Labeling MRI in Healthy Subjects,” Frontiers in Aging Neuroscience 10 (2018): 214. 30065646 10.3389/fnagi.2018.00214 PMC6056623

[12] C. Habeck , N. L. Foster , R. Perneczky , et al., “Multivariate and Univariate Neuroimaging Biomarkers of Alzheimer's Disease,” Neuroimage 40 (2008): 1503–1515. 18343688 10.1016/j.neuroimage.2008.01.056 PMC2441445

[13] P. Spetsieris , Y. Ma , S. Peng , et al., “Identification of Disease‐Related Spatial Covariance Patterns Using Neuroimaging Data,” Journal of Visualized Experiments: JoVE 76 (2013): e50319. 10.3791/50319 PMC3728991 23851955

[14] T. R. Melzer , R. Watts , M. R. MacAskill , et al., “Arterial Spin Labelling Reveals an Abnormal Cerebral Perfusion Pattern in Parkinson's Disease,” Brain 134 (2011): 845–855. 21310726 10.1093/brain/awq377 PMC3105489

[15] S. K. Meles , M. Pagani , D. Arnaldi , et al., “The Alzheimer's Disease Metabolic Brain Pattern in Mild Cognitive Impairment,” Journal of Cerebral Blood Flow and Metabolism 37 (2017): 3643–3648. 28929833 10.1177/0271678X17732508 PMC5718332

[16] F. Fazekas , J. Chawluk , A. Alavi , et al., “MR Signal Abnormalities at 1.5 T in Alzheimer's Dementia and Normal Aging,” American Journal of Roentgenology 149 (1987): 351–356. 3496763 10.2214/ajr.149.2.351

[17] North American Symptomatic Carotid Endarterectomy Trial , “Methods, Patient Characteristics, and Progress,” Stroke; A Journal of Cerebral Circulation 22 (1991): 711–720. 10.1161/01.str.22.6.711 2057968

[18] D. C. Alsop , J. A. Detre , X. Golay , et al., “Recommended Implementation of Arterial Spin‐Labeled Perfusion MRI for Clinical Applications: A Consensus of the ISMRM Perfusion Study Group and the European Consortium for ASL in Dementia,” Magnetic Resonance in Medicine 73 (2015): 102–116. 24715426 10.1002/mrm.25197 PMC4190138

[19] D. M. Garcia , G. Duhamel , and D. C. Alsop , “Efficiency of Inversion Pulses for Background Suppressed Arterial Spin Labeling,” Magnetic Resonance in Medicine 54 (2005): 366–372. 16032674 10.1002/mrm.20556

[20] H. J. Mutsaerts , J. Petr , L. Vaclavu , et al., “The Spatial Coefficient of Variation in Arterial Spin Labeling Cerebral Blood Flow Images,” Journal of Cerebral Blood Flow and Metabolism 37 (2017): 3184–3192. 28058975 10.1177/0271678X16683690 PMC5584689

[21] N. M. Hirsch , V. Toth , A. Forschler , et al., “Technical Considerations on the Validity of Blood Oxygenation Level‐Dependent‐Based MR Assessment of Vascular Deoxygenation,” NMR in Biomedicine 27 (2014): 853–862. 24809665 10.1002/nbm.3131

[22] K. Mouridsen , M. B. Hansen , L. Ostergaard , et al., “Reliable Estimation of Capillary Transit Time Distributions Using DSC‐MRI,” Journal of Cerebral Blood Flow and Metabolism 34 (2014): 1511–1521. 24938401 10.1038/jcbfm.2014.111 PMC4158667

[23] M. Joliot , G. Jobard , M. Naveau , et al., “AICHA: An Atlas of Intrinsic Connectivity of Homotopic Areas,” Journal of Neuroscience Methods 254 (2015): 46–59. 26213217 10.1016/j.jneumeth.2015.07.013

[24] X. Sun and W. Xu , “Fast Implementation of DeLong's Algorithm for Comparing the Areas Under Correlated Receiver Operating Characteristic Curves,” IEEE Signal Processing Letters 21 (2014): 1389–1393.

[25] J. Schroder , M. Heinze , M. Gunther , et al., “Dynamics of Brain Perfusion and Cognitive Performance in Revascularization of Carotid Artery Stenosis,” NeuroImage: Clinical 22 (2019): 101779. 30903966 10.1016/j.nicl.2019.101779 PMC6431743

[26] L. Zarrinkoob , A. Wahlin , K. Ambarki , et al., “Blood Flow Lateralization and Collateral Compensatory Mechanisms in Patients With Carotid Artery Stenosis,” Stroke; A Journal of Cerebral Circulation 50 (2019): 1081–1088. 10.1161/STROKEAHA.119.024757 PMC6485302 30943887

[27] A. E. Cavanna and M. R. Trimble , “The Precuneus: A Review of Its Functional Anatomy and Behavioural Correlates,” Brain 129 (2006): 564–583. 16399806 10.1093/brain/awl004

[28] S. He , Z. Liu , Z. Xu , et al., “Brain Functional Network in Chronic Asymptomatic Carotid Artery Stenosis and Occlusion: Changes and Compensation,” Neural Plasticity 2020 (2020): 9345602. 33029129 10.1155/2020/9345602 PMC7530486

[29] F. Fischer , C. Malherbe , E. Schlemm , et al., “Intrinsic Functional Brain Connectivity Is Resilient to Chronic Hypoperfusion Caused by Unilateral Carotid Artery Stenosis,” NeuroImage: Clinical 34 (2022): 103014. 35483135 10.1016/j.nicl.2022.103014 PMC9125779

[30] D. J. Wang , Y. Chen , M. A. Fernandez‐Seara , et al., “Potentials and Challenges for Arterial Spin Labeling in Pharmacological Magnetic Resonance Imaging,” Journal of Pharmacology and Experimental Therapeutics 337 (2011): 359–366. 21317356 10.1124/jpet.110.172577 PMC3083105

[31] N. S. Hartkamp , E. T. Petersen , M. A. Chappell , et al., “Relationship Between Haemodynamic Impairment and Collateral Blood Flow in Carotid Artery Disease,” Journal of Cerebral Blood Flow and Metabolism 38 (2018): 2021–2032. 28776469 10.1177/0271678X17724027 PMC6238174

[32] L. Schmitzer , S. Kaczmarz , J. Gottler , et al., “Macro‐ and Microvascular Contributions to Cerebral Structural Alterations in Patients With Asymptomatic Carotid Artery Stenosis,” Journal of Cerebral Blood Flow and Metabolism 44 (2024): 1629–1642. 38506325 10.1177/0271678X241238935 PMC11418673

[33] J. G. Woods , E. Achten , I. Asllani , et al., “Recommendations for Quantitative Cerebral Perfusion MRI Using Multi‐Timepoint Arterial Spin Labeling: Acquisition, Quantification, and Clinical Applications,” Magnetic Resonance in Medicine 92 (2024): 469–495. 38594906 10.1002/mrm.30091 PMC11142882

